# Cancer cell invasion alters the protein profile of extracellular vesicles

**DOI:** 10.1002/jex2.124

**Published:** 2023-11-27

**Authors:** Jens C. Luoto, Leila S. Coelho‐Rato, Cecilia Jungarå, Sara H. Bengs, Jannica Roininen, John E. Eriksson, Lea Sistonen, Eva Henriksson

**Affiliations:** ^1^ Faculty of Science and Engineering, Cell Biology Åbo Akademi University Turku Finland; ^2^ Turku Bioscience Centre University of Turku and Åbo Akademi University Turku Finland

**Keywords:** 3D cultures, EV heterogeneity, EV proteome, extracellular vesicles, invasion, PC3 cells, prostate cancer

## Abstract

Extracellular vesicles (EVs) are important mediators of intercellular communication involved in local and long‐range signalling of cancer metastasis. The onset of invasion is the key step of the metastatic cascade, but the secretion of EVs has remained unexplored at that stage due to technical challenges. In this study, we present a platform to track EVs over the course of invasive development of human prostate cancer cell (PC3) tumoroids utilizing in vivo‐mimicking extracellular matrix‐based 3D cultures. Using this EV production method, combined with proteomic profiling, we show that PC3 tumoroids secrete EVs with previously undefined protein cargo. Intriguingly, an increase in EV amounts and extensive changes in the EV protein composition were detected upon invasive transition of the tumoroids. The changes in EV protein cargo were counteracted by chemical inhibition of invasion. These results reveal the impact of the tumoroids’ invasive status on EV secretion and cargo, and highlight the necessity of in vivo‐mimicking conditions for uncovering novel cancer‐derived EV components.

## 1 INTRODUCTION

The leading cause of cancer‐related deaths worldwide is metastasis, where cancer cells break free from the primary tumour and invade the adjacent tissues. This is followed by cancer cell intravasation into the circulatory system and extravasation into new tissues, thereby facilitating the formation of secondary tumours (Hanahan & Weinberg, 2011). It is crucial to detect tumours before the onset of invasion, to be able to prevent the metastatic cascade and reduce cancer mortality. Tumour progression and metastasis depend strongly on intercellular communication, in which extracellular vesicles (EVs) play a key role by shuttling biomolecules, such as proteins, lipids and nucleic acids across cells and tissues (Yáñez‐Mó et al., 2015). Cells secrete EVs of different sizes, such as exosomes (30–100 nm), microvesicles (50–1000 nm) and larger cancer‐specific oncosomes (up to 10 µm) (Jeppesen et al., 2023). Cancer‐derived EVs have been shown to promote metastasis by increasing angiogenesis, suppressing the immune system, reprogramming fibroblasts and preparing pre‐metastatic niches in specific organs (Lucotti et al., 2022). EVs have evoked great interest as biomarkers for early detection of cancer, since they bear distinct cargo components and are detectable in all types of human biofluids, such as blood or urine (Bamankar & Londhe, 2023; Irmer et al., 2023). However, biofluids contain heterogeneous and complex populations of EVs secreted by different types of cells, which makes patient samples prone to major biological variations. Therefore, simulating the dynamic tumour development in a more controlled context is acutely needed for cancer EV research.

Previous studies on cancer EVs have extensively used conventional two‐dimensional (2D) cell cultures (Xu et al., 2018), in which in vivo tumour growth and development are poorly recapitulated (Duval et al., 2019). In contrast, three‐dimensional (3D) cell culture models provide a more accurate representation of tumour characteristics, allowing cancer cells to acquire similar morphology, growth and differentiation patterns as those detected in tumours (Duval et al., 2019). It is therefore no surprise that multiple studies have shown substantial differences in the secretion and cargo components of EVs between 2D and 3D cancer cell cultures (Al Hrout et al., 2023; Eguchi et al., 2018; Giusti et al., 2022; Kyykallio et al., 2022; Martins et al., 2023; Millan et al., 2022; Rocha et al., 2019; Szvicsek et al., 2019; Thippabhotla et al., 2019; Yang et al., 2020). These studies employed synthetic hydrogels and scaffolds, as well as extracellular matrix (ECM)‐based 3D cultures, but it remains to be shown whether EV secretion and characteristics change during tumour development and invasive transition.

In this study, we employed ECM‐based 3D cultures to investigate the secretion of EVs during the invasive development of tumoroids formed by human PC3 prostate cancer cells. Through this experimental setup and proteomic profiling, we discovered previously undefined protein content in EVs derived from prostate cancer cells. Moreover, we found a significant increase in EV amounts and substantial changes in their protein composition during the tumoroids’ transition to an invasive state. Importantly, these changes in EV protein content were abolished when invasion was chemically inhibited. Our study demonstrates that EV secretion of cancer tumoroids in 3D in vivo‐mimicking conditions is a dynamic process that changes according to the invasive state of the cells.

## 2 MATERIALS AND METHODS

### 2.1 MISEV2018 statement

This work was performed following the minimal information for studies of EVs 2018 (MISEV2018) guidelines (Théry et al., 2018). We have submitted all relevant data of our experiments to the EV‐TRACK knowledgebase (EV‐TRACK ID: EV200156) (Van Deun et al., 2017).

### 2.2 3D cell culture

To collect EVs secreted by human PC3 prostate cancer cells (CRL‐1435, ATCC) grown in 3D cultures, we utilized a Matrigel (354230, Corning) sandwich 3D culture system. In brief, an acid‐treated (HNO_3_) sterile 13 mm coverslip was placed into each well of a 24‐well plate (662160, Greiner). This enabled an even distribution of Matrigel and made the cultures portable and accessible for downstream imaging assays. The plate was chilled and kept cool on a cooling pad during the Matrigel application to ensure even polymerization across the plate. For Matrigel concentration optimization, Matrigel was diluted (3.5, 5.0 and 6.5 mg/mL) in ice‐cold serum‐free RPMI media (R5886, Merck, with 100 U/µg/mL penicillin/streptomycin [P/S, P0781, Merck] and 2 mM L‐glutamine [L‐glut, X0550, Biowest]). The Matrigel solutions (50 µL) were drop‐casted on the coverslips and the plates were incubated at 37°C, 5% CO_2_ for 1 h, during which Matrigel polymerized. PC3 cells (p6‐p16) were trypsinized and 5000 cells, counted with Countess II cell counter (Thermo Fisher Scientific), were suspended in 30 µL of EV‐free RPMI media (10% exosome‐depleted fetal bovine serum [FBS, A2720801, Thermo Fisher Scientific], 100 U/µg/mL P/S, and 2 mM L‐glut) and seeded on each coverslip on top of the Matrigel. The cells were allowed to attach to Matrigel for 1 h in an incubator (37°C, 5% CO_2)_, where after the excess media was removed. A top layer of 30 µL Matrigel (3.5, 5.0 and 6.5 mg/mL) was applied and allowed to polymerize for 1 h in the incubator. The wells were then filled with 600 µL of EV‐free RPMI media and the plates were placed back in the incubator.

### 2.3 EV harvest from 3D cultures

The conditioned media was carefully harvested every 2 days (48 h) and replaced with fresh EV‐free RPMI media. The media was collected in 15 mL (188271, Greiner) or 50 mL tubes (210261, Greiner) and was centrifuged twice at 300 × *g* for 10 min at 4°C in a Sigma 4‐16KS centrifuge (Sigma Laboratory Centrifuges) to remove the cells, and the supernatant was stored at −80°C until use.

### 2.4 EV harvest from Dissolved Matrigel (DM)

To collect EVs trapped inside of Matrigel, the matrix was dissolved at the end of the 3D culture, and the cells were removed. For this purpose, the Matrigel layers were scraped with a scalpel into 8 mL of Gentle Cell Dissociation Reagent (GCDR, 07174, Stemcell) and incubated for 15 min at 37°C, after which the suspension was mixed by pipetting and placed on a revolver for 15 min at room temperature (RT). To separate the cells from the dissolved Matrigel, the suspensions were centrifuged at 300 × *g* for 10 min at RT. The clear supernatant was collected and the remaining cell‐Matrigel pellet was dissolved in 14 mL of phosphate‐buffered saline (PBS, L0615, Biowest) and centrifuged at 300 × *g* for 10 min. The supernatants were pooled together as the DM sample. To remove any remaining cells from the sample, the solution was centrifuged twice at 300 × *g* for 10 min at 4°C. The supernatant and cell pellets were stored at −80°C until used for EV isolation and cell viability assessments, respectively.

### 2.5 EV harvest from 2D cell culture

PC3 cells (p9‐p16) were plated onto two 15 cm plates in RPMI media (10% FBS [S181B, Biowest], 100 U/µg/mL P/S and 2 mM L‐glut) and were grown in 37°C, 5% CO_2_ until they reached 60% confluency. The cells were then washed with PBS and 15 mL EV‐free RPMI (10% exosome‐depleted FBS, 100 U/µg/mL P/S and 2 mM L‐glut) was added. After 48 h, cell viability of over 95% was confirmed by Trypan blue staining and the conditioned media was collected and processed as described for the 3D cultures.

### 2.6 EV isolation by differential centrifugation

The EV samples were isolated by differential centrifugation as previously described (Kowal et al., 2016). In brief, the samples were thawed at RT and transferred to centrifuge tubes (357003, Beckman Coulter) and centrifuged at 10,000 × *g* in an Avanti J‐26 XPI centrifuge (Beckman Coulter) using a JA‐25,50 rotor (k‐factor 2143.7) for 40 min at 4°C. The supernatant was transferred to new centrifuge tubes (331372 or 326823, Beckman Coulter) and centrifuged for 90 min at 4°C in an Optima L‐90K ultracentrifuge (Beckman Coulter) at 28,000 rpm (∼97,000 × *g*) using an SW41 Ti rotor (k‐factor 266). For larger volumes, an SW 32 Ti rotor was used at 29,000 rpm (∼103,000 × *g*, k‐factor 248). The pellet from 10,000 × *g* centrifugation (10K) was washed with PBS and centrifuged again at 10,000 × *g* for 40 min at 4°C. The supernatants were discarded and the pellets (10K samples) were dissolved in 100 µL PBS and frozen at −80°C. The 100K samples’ supernatants were discarded and the pellets were washed with PBS and centrifuged again at 100,000 × *g* for 90 min at 4°C. The supernatants were discarded and the pellets (100K samples) were re‐suspended in 100 µL PBS and frozen at −80°C.

### 2.7 Nanoparticle tracking analysis (NTA) of the EV samples

NTA was performed at the EV Core Facility at University of Helsinki using a NanoSight LM14C (Malvern Panalytical) equipped with blue (404 nm, 70 mW) laser and SCMOS camera to characterize particles between 10 and 1000 nm in the EV samples. The samples were diluted in 0.1 µm filtered (Millex VV, Millipore) PBS to obtain 40–100 particles/view, and five 30 s videos were recorded using camera level 14. The data were analysed using NTA software 3.0 with the detection threshold 3–4 and screen gain at 10. All NTA experiments were repeated with at least three biological replicates.

### 2.8 Zeta potential (ZP) measurements of the EV samples

A ZetaView PMX‐120 was used to analyse the ZP of EVs diluted in PBS. The data were acquired with sensitivity 85, shutter at 100, max. area at 1000, min. area at 10, min. brightness at 30 and max. brightness at 255. Each measurement was run for three cycles.

### 2.9 Immunoblotting

For the EV samples used in Figures 2c and 3b, equal volumes of the EV samples were lysed in RIPA lysis buffer (150 mM NaCl, 1% Triton X‐100, 0.5% sodium deoxycholate, 0.1% SDS, 50 mM Tris‐HCl,pH 8.0, supplemented with 1x protease inhibitor cocktail [04693159001, Roche] and 0.5 mM phenylmethylsulfonyl fluoride [PMSF]), on ice for 1 h and then supplemented with 3x Laemmli sample buffer with or without 2‐mercaptoethanol according to antibody preferences. The EV samples used in Figure 5 and 6c were lysed in the same way, but their protein concentration was determined by Pierce Micro BCA (23235, Thermo Scientific) or BCA protein assay kit (23225, Thermo Scientific), and equal protein amounts were used for the immunoblots. Cells were lysed in RIPA lysis buffer on ice for 1 h followed by centrifugation at 20,000 × *g* to remove the insoluble fraction. The protein concentration was measured and 1–5 μg of lysates were used as samples. All samples were boiled for 5 min and resolved by SDS‐PAGE on 4%–20% Bio‐Rad (561094) or Nippon Genetics (PG‐S420) gradient gels. After transferring onto a nitrocellulose membrane, the membranes were blocked with 5% fat‐free milk dissolved in 0.05% PBST for 1 h. The membranes were rinsed with MQ and incubated overnight with primary antibodies (diluted in either PBS or TBS with 0.5% BSA and 0.02% NaN_3_) at 4°C, followed by three PBST or TBST 0.3% washes and incubation in HRP‐conjugated secondary antibodies in blocking solution at room temperature for 1 h. After additional three washes, the signals were detected with enhanced chemiluminescence (34579 and 34094, Thermo Fisher Scientific) and an iBright FL1000 imager (Thermo Fisher Scientific). All immunoblotting experiments were repeated at least three times.

### 2.10 Antibodies

#### 2.10.1 Primary antibodies

Anti‐TSG101 (ab125011, 1:1000), anti‐GM130 (ab52649, 1:1000), anti‐LAMA 1&2 (ab7463, 1:1000), anti‐CD59 (ab133707, 1:1000) and anti‐GAPDH (ab9485, 1:2500) were purchased from Abcam.

Anti‐CD81 (MA5‐13548, 1:500) was bought from ThermoFisher Scientific.

Anti‐CD63 (CBL553, 1:1000) was acquired from Merck.

Anti‐PARP1 (sc‐8007, 1:500) anti‐FGB (sc‐271017, 1:500), anti‐APOA1 (sc‐376818, 1:100), anti‐ITGB4 (sc‐514426, 1:300) and anti‐VCL (sc‐73614, 1:500) were bought from Santa Cruz Biotechnology.

Anti‐LMNA (4777, 1:1000) and anti‐CD73 (13160, 1:500) were obtained from Cell Signaling Technology.

Anti‐HSC70 (ADI‐SPA‐815, 1:1000) was purchased from Enzo Life Sciences.

#### 2.10.2 Secondary antibodies

Anti‐Rabbit IgG (H+L), HRP Conjugate (W4011, 1:5000), and anti‐Mouse IgG (H+L), HRP Conjugate (W4021, 1:5000) were purchased from Promega.

Anti‐Mouse IgG2a heavy chain (HRP) (ab97245, 1:5000), anti‐Mouse IgG2b heavy chain (HRP) (ab97250, 1;5000) and anti‐Rat IgG2a H&L (HRP) (ab106783, 1:5000) was bought from Abcam.

### 2.11 EV isolation by OPTI‐prep

In order to obtain enough material for the subsequent assays, the 3D culture was extended to 14 days, and conditioned media was collected and pooled from days 2–8 and days 10–14. The pooled media and DM samples were concentrated using a Centricon Plus‐70 10K filter (UFC701008, Merck) and diluted to 1 mL using PBS. The concentrates were then layered on top of a high‐resolution iodixanol density gradient as previously described (Van Deun et al., 2014). In brief, solutions of 5%, 10%, 20% and 40% iodixanol were made by mixing appropriate amounts of a homogenization buffer (0.25 M sucrose, 1 mM EDTA, 10 mM Tris‐HCL, pH 7.4) and an iodixanol working solution. This working solution was prepared by combining a buffer (0.25 M sucrose, 6 mM EDTA, 60 mM Tris‐HCl, pH 7.4) and a stock solution of OptiPrep™ (60% (w/v) aqueous iodixanol solution, D1556, Merck). The gradient was formed by layering 2.4 mL of 40%, 20%, 10% and 5% solutions on top of each other in a 13.2 mL open‐top polyallomer tube (331372, Beckman Coulter). The 1 mL concentrates were overlaid on top of the gradient, and centrifuged at 100,000 × *g* (28,000 rpm, k‐factor 266) in an Optima L‐90K ultracentrifuge (Beckman Coulter) using an SW41 Ti rotor for 18 h at 4°C with slow deacceleration. The gradient was separated from the top into 1 mL fractions, diluted to 11 mL with PBS, and centrifuged at 100,000 × *g* for 3 h at 4°C with max deacceleration. The acquired pellets were diluted in 200–500 µL of PBS, split into smaller aliquots for downstream assays, and stored at −80°C.

### 2.12 EV lysis, in‐solution digestion, and LC‐MS/MS

Isolated EVs were lysed in a buffer containing 8 M urea, 0.5% NP40, 2 mM EDTA, 150 mM NaCl and one tablet of protease and phosphatase inhibitors (A32959, Thermo Fisher Scientific) for 30 min on ice, the samples were sonicated for 5 min (30´ on and 30´ off) and the proteins were precipitated using 4–5 volumes of cold acetone overnight at −20°C. Samples were cleared by centrifugation for 15 min using 16,000 × *g* at 4°C and the pellet was dissolved in 100 µL of 6 M urea in 25 mM ammonium bicarbonate. Approximately 100 µg of protein was used for in‐solution digestion. The samples were reduced with 10 mM DTT for 1 h at 37°C and alkylated with 40 mM iodoacetamide for 1 h in the dark. The alkylation was quenched with 40 mM DTT and the urea concentration was diluted by adding 900 µL of 25 mM ammonium bicarbonate. Sequencing grade trypsin was added to each sample in a 1:30 ratio to total protein and the samples were incubated overnight at 37°C. The peptides were acidified with TFA and were desalted using Sep Pak C18 columns (100 mg, Waters) according to the instructions of the manufacturer. The samples were dried on a speed‐vac centrifuge and stored at −80°C. Before analysis, the samples were dissolved in 0.1% formic acid and approximately 100 ng of each sample was loaded for the LC‐MS/MS analysis using an Easy‐nLC 1000 liquid chromatograph (Thermo Fisher Scientific) coupled to an Orbitrap Fusion Lumos Tribrid Mass Spectrometer (Thermo Fisher Scientific). The peptides were loaded on a pre‐column (100 µm × 2 cm), followed by separation in an analytical column (75 µm × 15 cm), both packed with 5 µm ReproSil‐Pur 200 Å C18 silica particles (Dr. Maisch HPLC GmbH). The peptides were separated using a 60 min gradient (5%–42% B in 50 min, 42%–100% B in 6 min, 100% B for 4 min, in which solvent B was 80% acetonitrile 0.1% formic acid in water) at a flow rate of 300 nL/min. MS/MS data were acquired in positive ionization mode using data‐dependent acquisition using a 2.5 s cycle time. The MS survey scans were acquired with a resolution of 120,000 with the range of 300−1700 m/z, an AGC target of 7.0 e5, and a maximum injection time of 50 ms. The ions were selected for HCD fragmentation using an isolation window of 1.6 m/z, with AGC target of 104 and a maximum injection time of 50 ms. MS/MS spectra were recorded with 30,000 resolution, and a dynamic exclusion window of 35 s was used.

### 2.13 Proteomic data processing

Raw files obtained from the LC‐MS/MS analyses were processed using MaxQuant software (version 1.6.7.0) (Tyanova, Temu, & Cox, 2016) and searched against a SwissProt human protein database (https://www.uniprot.org/, release 20/10/2019) with added common contaminants using the built‐in Andromeda search engine. Trypsin digestion with a maximum of two missed cleavages, cysteine carbamidomethylation as a fixed modification, and methionine oxidation as variable modification were selected as the parameters of these searches. “Match between runs” option in MaxQuant was used with a matching time window of 2 min and an alignment time window of 20 min. The peptide level false discovery rate (FDR) was set to 1% and was determined by searching against a concatenated normal and reversed sequence database.

Label‐free quantitation was performed using the fast LFQ algorithm. Otherwise, the default settings in MaxQuant were used in data processing. The database search results with LFQ intensities were analysed using Perseus (version 1.6.7.0) (Tyanova, Temu, Sinitcyn, et al., 2016). Proteins only identified by site, proteins considered contaminants and reverse sequences were discarded. Proteins identified with less than two unique peptides were filtered out and only proteins with two values in at least one group were kept. Missing values were not imputed. The results are represented as log2 (LFQ) of two independent replicates. Venn diagrams were made using Funrich (Pathan et al., 2015) (last updated 11.11.2020). Table S1 contains all the identifications and transformed LFQ values.

### 2.14 Transmission electron microscopy (TEM)

TEM sample preparation and imaging were performed by the EV Core Facility, University of Helsinki, essentially as previously described (Puhka et al., 2017). In brief, pioloform‐ and carbon‐coated copper grids were glow discharged before the samples were loaded. The grids and samples were fixed with 2% paraformaldehyde in 0.1 M NaPO_4_ buffer (pH 7.0), stained with 2% neutral uranyl acetate, and further stained and embedded in uranyl acetate and methyl cellulose mixture (1.8/0.4%). The EVs were viewed using Jeol JEM‐1400 (Jeol Ltd) operating at 80 kV. Images were acquired with Gatan Orius SC 1000B CCD‐camera (Gatan Inc.).

### 2.15 Statistics and reproducibility

All experiments were repeated with at least three biological repeats unless otherwise stated. Data are expressed as mean ± SEM. For multiple group comparisons, one‐way ANOVA or two‐way ANOVA, followed by a Tukey test, was performed. A *p*‐value <0.05 was considered statistically significant. GraphPad Prism 8 was used for statistical analysis.

### 2.16 3D culture cell counting

After harvesting media from 3D cultures, coverslips were collected and placed in 14 mL of ice‐cold PBS supplemented with 5 mM of EDTA. The suspensions were mixed by rotation for 45 min at 4°C to fully dissolve the Matrigel, followed by centrifugation at 200 × *g* for 5 min at 4°C. The supernatant was removed and the cells were washed with 14 mL cold PBS, mixed, and recentrifuged at 200 × *g* for 5 min at 4°C. The supernatant was removed and the cell pellets were diluted in 2 mL trypsin (L0931, Biowest) (37°C) and incubated for 15 min at 37°C, agitating the tubes every few minutes. After the incubation, 10 mL of RPMI (10% FBS, 100 U/µg/mL P/S, and 2 mM L‐glut) was added to the tubes and the cells were pelleted by 5 min centrifugation at 300 × *g* at RT. The cells were resuspended in 100 µL of media and counted using the Countess II cell counter (Thermo Fisher Scientific). Four wells were counted for each time point to obtain an average cell amount per well. The experiments were repeated five times.

### 2.17 Brightfield imaging of 3D cultures

The 3D cultures were imaged from multiple wells every 48 h using a ZEISS Axio Vert.A1 microscope with an Axiocam 506 camera and an LD A‐Plan 5x/0.15 Ph1 objective. Brightness and contrast were adjusted using ZEN 2012 (ZEISS). Adjustments were applied across the entire images without loss of data.

### 2.18 IPA‐3 treatment of 3D cultures

Using our 3D culture setup, PC3 cells were grown as tumoroids in Matrigel and imaged every 48 h. On day 4 onward the media was changed to contain either 15 µM of IPA‐3 (I2285, Sigma‐Aldrich, dissolved in DMSO) or the equivalent volume of DMSO (D8418, Sigma‐Aldrich) as a control. The cells were washed with PBS after each EV harvest. The collected media from days 2 to 8 and days 10 to 14 were pooled together and EVs were isolated using OPTI‐prep density gradient fractionation. The cells were collected and lysed after day 14. All samples were analysed by immunoblotting.

## 3 RESULTS

### 3.1 In vivo‐mimicking ECM‐based 3D cultures enable EV isolation during invasive transition of tumoroids

**FIGURE 1:**
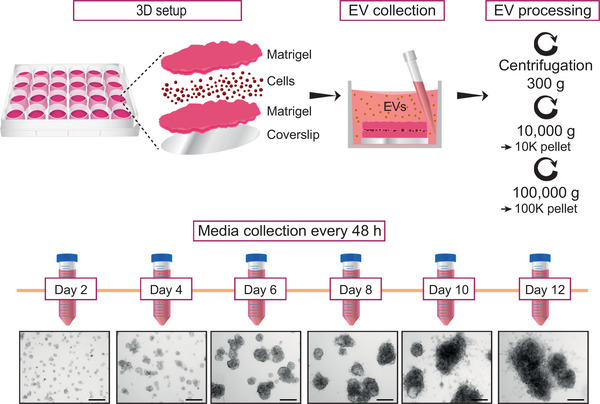
Isolation of EVs from ECM‐based 3D cultures undergoing invasive transition. Graphical workflow of EV isolation from the ECM‐based 3D cell culture. PC3 cells were seeded on top of coverslips in‐between two layers of Matrigel (3.5 mg/mL) and allowed to grow and form tumoroids during 12 days. Conditioned media of the tumoroid cultures was collected every two days, from which EVs were isolated by differential centrifugation into 10 and 100K pellets. Shown are representative brightfield images of the tumoroids. Scale bars 200 µm.

To address if the invasive development of tumours affects the secretion and characteristics of EVs, we established an in vivo‐mimicking EV production method using 3D cultures undergoing invasive transition (Figure 1). We seeded human PC3 prostate cancer cells between two layers of the ECM‐based hydrogel Matrigel and allowed the cells to divide and form tumoroids. The tumoroid‐derived EVs were collected from the conditioned media every other day and the EVs were isolated by differential centrifugation into 10 and 100K pellets. The tumoroids underwent dynamic phenotypic changes during the culture period of 12 days. A major phenotypic change was detected from day 10 onwards, as cells invaded the matrix surrounding the tumoroid, forming clearly visible invasive structures (Figure 1). The timing of the invasive transition is in agreement with previous studies conducted with PC3 cells (Björk et al., 2016; Härmä et al., 2010).

Optimal conditions for EV isolation and PC3 tumoroid development were tested with three different Matrigel concentrations (3.5, 5.0 and 6.5 mg/mL) (Figure S1A). In all cases, PC3 cells invaded the surrounding ECM and the cultures produced particles with similar sizes and amounts, as determined by NTA (Figure S1B–D). The highest quantity of secreted particles was found in 3.5 mg/mL of Matrigel (Figure S1C), and this concentration was chosen for all assays.

### 3.2 The invasive transition of prostate cancer tumoroid causes a surge in EV secretion and a change in surface characteristics

**FIGURE 2:**
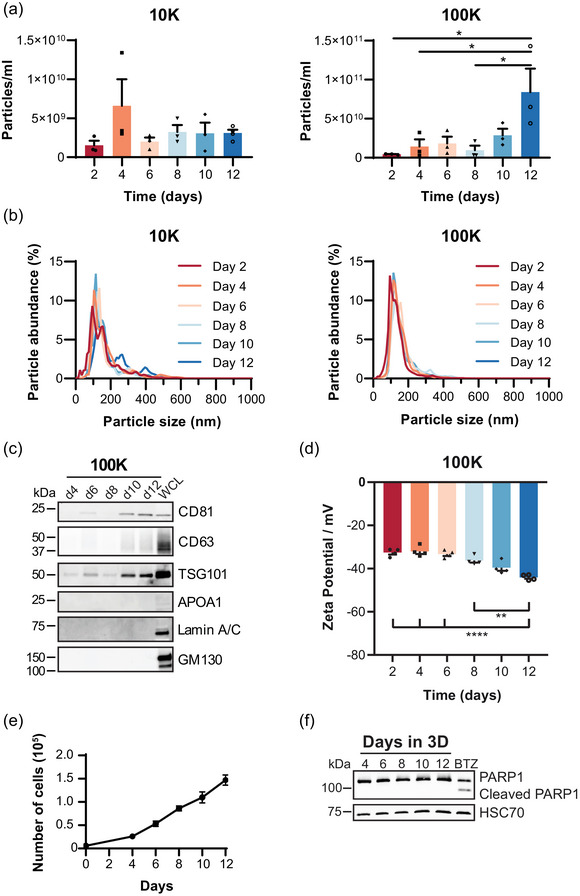
EV secretion increases substantially at the time of invasive transition of PC3 tumoroids. (a) Particle concentration of the 10 and 100K pellets obtained by nanoparticle tracking analysis (NTA). Significant differences were assessed by one‐way ANOVA with a Tukey post hoc test, *< 0.05, +SEM, *n* = 4. (b) EV size distribution of the 10 and 100K pellets detected by NTA shown as mean values of four independent experiments. (c) Immunoblot analysis of EV markers CD81, CD63 and TSG101 in 100K pellets. APOA1, Lamin A/C and GM130 were used as purity controls for the EV samples (d4‐d12). WCL, whole cell lysate. (d) ZETA potential measurement of the 100K samples. Significant differences were assessed by one‐way ANOVA with a Tukey post hoc test, *< 0.05, +SEM, *n* = 4. (e) Number of PC3 cells grown per well as described in Figure 1. The culture was started (day 0) with 6000 cells per well. Mean values of five independent repeats are shown +SEM. (f) Immunoblot analysis of PARP1 in PC3 cells grown as in Figure 1. Bortezomib (BTZ) treatment (300 nM, 22 h) of 2D grown PC3 cells was used as a positive control for PARP1 cleavage. HSC70 was used as a loading control.

To characterize the EVs secreted from the developing PC3 tumoroids undergoing invasive transition, the collected and isolated 10 and 100K EV pellets were analysed by NTA, immunoblotting, and zeta potential analyses (Figure 2a–d). While the particle sizes were similar throughout the time course in both 10 and 100K samples   , the particle concentration showed a significant increase at day 12 in the 100K samples (Figure 2a, 2b, S2). Furthermore, immunoblotting showed markedly increased levels of selected EV markers; CD81 antigen (CD81), CD63 antigen (CD63) and tumour susceptibility gene 101 protein (TSG101) at day 10 onwards (Figure 2c). No major sample contamination was detected, as neither lipoproteins, for example, apolipoprotein A1 (APOA1), nor intracellular proteins, for example, lamin A/C and Golgi matrix protein (GM130), were present in the EV samples. Zeta potential analysis of the EVs showed a gradual decrease in particle surface charge throughout the time course, with a significant change on day 12 compared to days 2–8 (Figure 2d). This change in zeta potential indicates that the molecular composition of the EV surfaces was modified during the tumoroid development. To address if the sudden rise in the EV secretion was caused by an abruptly increased number of cells, we counted the cells of the developing tumoroid cultures. Cells displayed a linear proliferation throughout the culture period of 12 days (Figure 2e), indicating that the surge in EV secretion was not due to an increase in cell number. Apoptotic bodies derived from dying cells could also explain the increasing amounts of particles produced by 3D cultures. However, no signs of apoptosis were detected in the tumoroids as assessed by cleavage of the apoptosis marker poly (ADP‐ribose) polymerase 1 (PARP1) with immunoblotting (Figure 2f).

Because Matrigel is a mixture of ECM proteins, it prompted us to examine whether Matrigel‐derived particles could interfere with EV analyses. For this purpose, we excluded cells from our experimental setup (Figure 1), and isolated particles from the culture media and the dissolved Matrigel. NTA results revealed that Matrigel released particles of similar size to cultures with PC3 cells (Figure S1D and S3A‐C), but their quantity was only 2%–3% compared to the amounts of particles from cell cultures (Figure S3D). Notably, these Matrigel particles were devoid of EVs, as confirmed by immunoblotting using EV markers TSG101, CD81 and CD63 (Figure S3E). Instead, laminin subunit alpha 1 (LAMA1) and 2 (LAMA2), two major components of Matrigel (Hughes et al., 2010), known to form large ECM complexes (Hamill et al., 2009), were detected. Thus, particles originating from Matrigel do not interfere with EV isolation from ECM‐based 3D cultures. In summary, our data shows that an increase in EV amount and a change in EV surface charge coincide with the invasive development of the PC3 tumoroids.

### 3.3 Proteomic profiling revealed previously undefined EV cargo secreted by PC3 tumoroids

**FIGURE 3:**
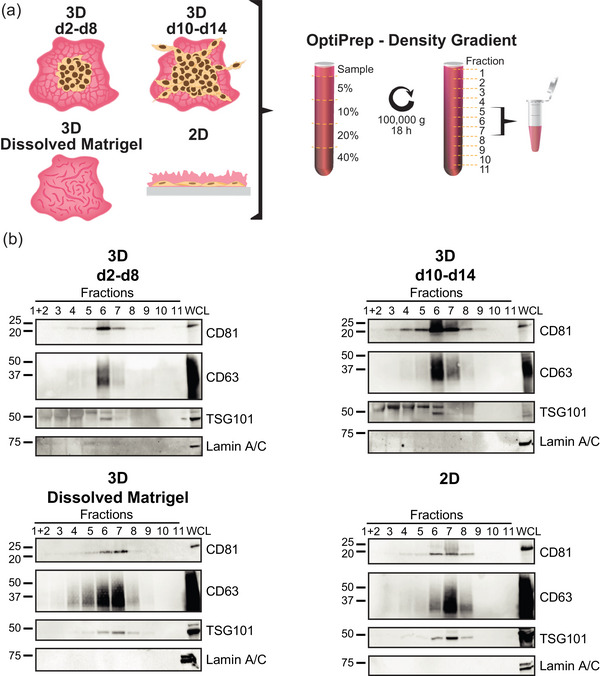
High‐resolution gradient purification of EVs from non‐invasive and invasive PC3 tumoroid cultures. (a) EVs were isolated from the conditioned media of non‐invasive PC3 3D cultures at days 2–8 (3D d2‐d8), invasive cultures at days 10–14 (3D d10‐d14), dissolved Matrigel at day 14 (3D Dissolved Matrigel), and conditioned media of 2D cultures (2D). OptiPrep density gradient centrifugation was employed for these samples and the resulting 11 fractions were screened for EVs (b). Fractions 5, 6 and 7 were pooled together and used for subsequent assays (Figures 4 and 5). (b) Immunoblot analyses of the 11 fractions with EV markers CD81, CD63 and TSG101. Lamin A/C was used as an EV purity marker and PC3 whole cell lysate (WCL) as a positive control for immunoblotting.

**FIGURE 4:**
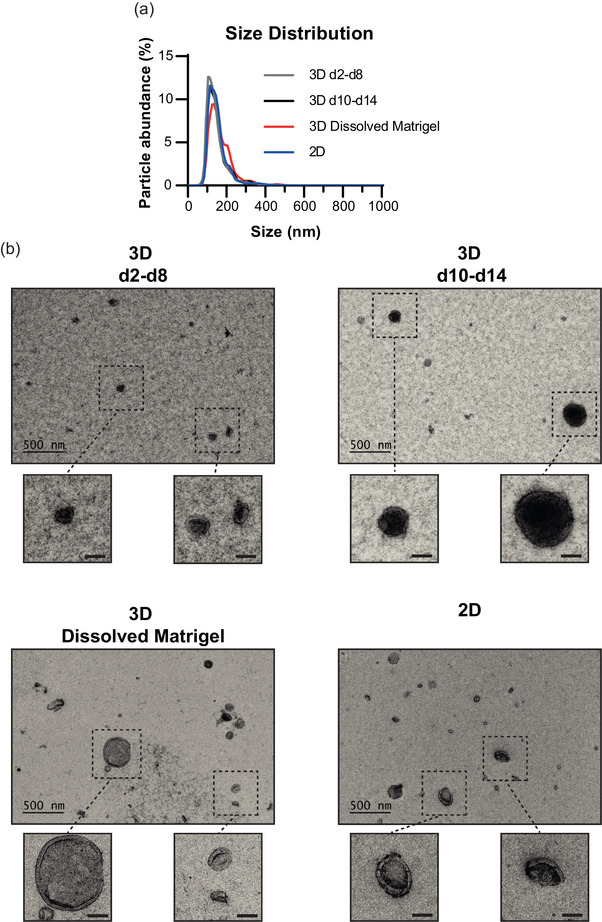
Non‐invasive and invasive PC3 tumoroid cultures secrete EVs of similar size and morphology. (a) Size distribution of EVs from non‐invasive (3D d2‐d8) and invasive (3D d10‐d14) 3D cultures, dissolved Matrigel (3D Dissolved Matrigel), and from 2D cultures (2D). Mean values of three independent experiments conducted with NTA are shown. (b) Morphology of the EVs from 3D and 2D cultures as analyzed by TEM. Representative images with scale bar 500 nm for the full sized and 100 nm for the zoomed in images.

To investigate the potential qualitative differences in the secreted EVs during the invasive development of PC3 tumoroids, we performed comprehensive analyses of the size, morphology and protein content using NTA, transmission electron microscopy (TEM), and liquid chromatography‐tandem mass spectrometry (LC‐MS/MS). To ensure minimal cell‐ or media‐derived contaminants in the EV samples, we employed the high‐resolution iodixanol gradient purification method, known for its improved EV sample purity compared to ultracentrifugation (Coumans et al., 2017). EVs were isolated from the conditioned media of non‐invasive (3D day 2–8) and invasive (3D day 10–14) cultures (Figure 3a). The cultures were expanded to day 14 to gain enough material, whereafter the Matrigel was dissolved to assess if EVs had been trapped therein. For comparison, EVs secreted by 2D PC3 cultures were included in the analyses. From the density gradient purification, 11 fractions were recovered, analysed by immunoblotting with the EV markers CD81, CD63 and TSG101 (Figure 3b), and the EV‐containing fractions 5–7 were pooled together for subsequent analyses. The NTA analyses showed that particle size distribution was similar in all samples, with particle mean sizes ranging between 140 and 169 nm (Figure 4a and Figure S4A). This size distribution is in line with the samples isolated with differential centrifugation method (Figure 2b). Furthermore, typical cup‐shaped EVs were detected by TEM (Figure 4b), indicating that regardless of the culture conditions, PC3 cells secrete EVs with no differences in size or shape.

**FIGURE 5:**
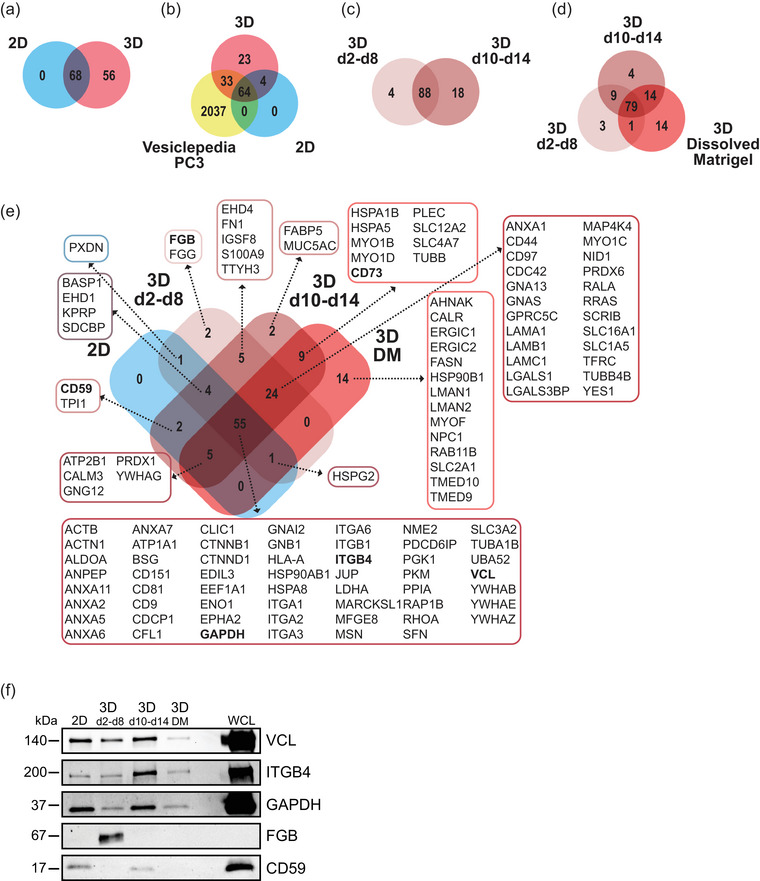
EV protein content changes upon invasive transition of 3D cultured PC3 cells. LC‐MS/MS analyses of EV proteins from non‐invasive PC3 3D cultures at days 2–8 (3D d2‐d8), invasive cultures at days 10–14 (3D d10‐d14), dissolved Matrigel at day 14 (3D Dissolved Matrigel), and 2D cultures (2D), *n* = 2. (a and b) Venn diagram of EV proteins from 2D and 3D cultures and with EV proteins deposited in the Vesiclepedia database for PC3 cells. (c) Venn diagrams of EV proteins from non‐invasive and invasive 3D cultures, and (d) EV proteins retained by the Matrigel (3D Dissolved Matrigel). (e) Venn diagram of the identified EV proteins. (f) Immunoblot analysis of the EV samples with selected MS identified proteins; VCL, ITGB4, GAPDH, FGB and CD59. DM, dissolved Matrigel; WCL, whole cell lysate of 3D grown cells.

The LC‐MS/MS analyses revealed extensive differences in protein cargo of EVs secreted by either 2D or 3D cultures (Figure 5a, Table S1). Among the 124 identified EV proteins, 56 were unique for 3D cultures, whereas 2D cultures contained no unique proteins (Figure 5a). Importantly, we identified 27 proteins that have not been previously registered in the Vesiclepedia database for EVs derived from PC3 cells (Kalra et al., 2012, Vesiclepedia accessed 26.7.2023) (Figure 5b). These results demonstrate that the cell culture conditions and isolation methods have a profound effect on EV cargo composition.

### 3.4 The invasive transition leads to major differences in EV protein composition

Our proteomic profiling also showed that EV protein content changed strikingly upon invasive transition of the PC3 tumoroids. The EVs secreted by the non‐invasive (3D d2‐d8) and invasive (3D d10‐d14) tumoroids had distinct protein contents, which became even more diversified upon invasion (Figure 5c). Of the identified 124 EV proteins secreted by the 3D cultures, four were unique for the non‐invasive and 18 for the invasive cultures. The EVs bound by the Matrigel (3D Dissolved Matrigel) contained 14 unique proteins (Figure 5d). A further examination of the identified proteins revealed that the cancer cells secrete different types of EV cargo proteins before and after invasion (Figure 5e). EVs secreted by invasive tumoroids (3D d10‐d14) included proteins promoting tumour growth (solute carrier family 12 member 2, SLC12A2; sodium bicarbonate cotransporter 3, SLC4A7) (Demian et al., 2019; Lee et al., 2016), tumour invasion (fatty acid binding protein 5, FABP5) (O'Sullivan & Kaczocha, 2020) and immunosuppression (cluster of differentiation 73, CD73) (Gao et al., 2014). Interestingly, these proteins were absent from EVs secreted by non‐invasive tumoroids (3D d2‐d8). In addition to the distinct EV proteins, all samples derived from 2D or 3D culture conditions contained a common set of classical EV proteins (Théry et al., 2018), consisting of tetraspanins, integrins, annexins and heat shock proteins among others (Figure 5e).

**FIGURE 6:**
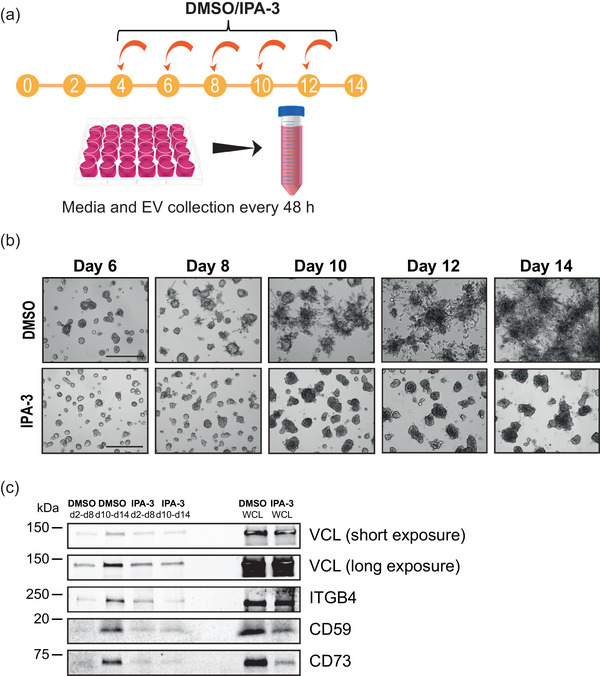
Inhibition of invasion changes the EV cargo content. (a) IPA‐3 treatment of PC3 cells grown in 3D. The media was harvested and replenished every 48 h. From day 4 onwards, 15 µM of IPA‐3 or the equivalent volume of DMSO was added to the media. Harvested media from days 2–8 and 10–14 were pooled together. (b) Representative brightfield images of the DMSO or IPA‐3 treated cells. Scale bars 200 µm. (c) Immunoblot analysis of EVs from DMSO or IPA‐3 treated PC3 cells with VCL, ITGB4, CD59 and CD73. WCL from DMSO and IPA‐3 treated tumoroids were harvested at day 14.

To verify the LC‐MS/MS results, a subset of the identified proteins was selected for immunoblot analysis (Figure 5f). The results conformed to the expression patterns of the proteins that were analysed by LC‐MS/MS. Vinculin (VCL), integrin β4 (ITGB4) and glyceraldehyde‐3‐phosphate dehydrogenase (GAPDH) were detected in all EV samples, which is consistent with the Vesiclepedia database. In contrast, fibrinogen beta chain (FGB) was only detected in the 3D d2‐d8 sample, while CD59 glycoprotein (CD59) was present in the 2D and 3D d10‐d14 samples, as was also shown by LC‐MS/MS. To address whether it was the invasive switch that caused the differential EV cargo loading, we treated the tumoroids with p21 activated kinase inhibitor IPA‐3 (Figure 6a), which is known to inhibit invasion of PC3 tumoroids (Björk et al., 2016). The IPA‐3 treatment blocked completely the formation of invasive structures, whereas the DMSO treated controls showed invasive morphology after day 8 (Figure 6b). Accordingly, VCL and ITGB4 amounts in EVs increased substantially from the d2‐d8 to d10‐d14 DMSO samples, but no increase occurred upon IPA‐3 treatment (Figure 6c). The inhibitory effect of IPA‐3 was even more prominent in the case of CD59 and CD73, as only a very weak signal was detected in the d10‐d14 EV sample. Taken together, these results demonstrate that the invasive transition of the PC3 cells has a major impact on the protein cargo composition of the secreted EVs.

## 4 DISCUSSION

EVs are a fundamental part of intercellular communication that cancer cells utilize to promote tumour growth and metastasis (Lucotti et al., 2022). However, how EVs’ secretion and characteristics change during tumour development and invasive transition has remained unknown. To test our hypothesis that cancer cells release distinct types of EVs over the course of invasive transition, we utilized an ECM‐based 3D culture method for studying secretion of EVs from PC3 prostate cancer tumoroids undergoing invasion. Previously used 3D culture models for EV studies have utilized synthetic hydrogels (Kyykallio et al., 2022; Millan et al., 2022; Thippabhotla et al., 2019), polymeric hard scaffolds (Eguchi et al., 2018; Martins et al., 2023), and scaffold‐free approaches (Giusti et al., 2022; Rocha et al., 2019), which allow the cells to form solid tumours, but impede invasion. In the ECM‐based hydrogel Matrigel, PC3 prostate cancer cells form tumoroids that over the course of the culture period invade the surrounding hydrogel, as shown in our previous study (Björk et al., 2016). Therefore, we chose to use Matrigel for EV production in 3D. Since Matrigel‐derived particles constituted only 2% of the total particles detected in our culture system (Figure S3D) and contained no EVs (Figure S3E), we concluded that Matrigel is suitable for EV production in 3D cultures.

Over the course of invasive development of the PC3 tumoroids we detected a significant increase in EV amounts after ten days in culture, which coincides with the invasive transition (Figures 1 and 2). The sizes of the secreted EVs did not change, as small EVs of 100–200 nm were secreted throughout the timespan of the 3D culture (Figure 2a,b), as well as from cells grown in 2D (Figure 4a,b). Furthermore, the surface charge of the EVs became significantly more negative at day 12 (Figure 2d). Changes in the zeta potential of EVs have been associated with various biomolecules, including lipids (Midekessa et al., 2020) and glycoproteins (Williams et al., 2019), which contribute to an increase in negative surface charge of the EVs. While our study focused on uncovering EV‐associated proteins, the zeta potential changes suggest that also other cargo components, such as lipids and nucleic acids, could be modified during invasion.

The assessment of the EVs’ qualitative differences with proteomic profiling revealed that PC3 cells grown in either 2D or 3D cultures secrete EVs with remarkably different protein cargo (Figure 5a), which is consistent with previous studies using other cancer cell lines (Eguchi et al., 2018; Giusti et al., 2022; Kyykallio et al., 2022; Millan et al., 2022; Rocha et al., 2019; Thippabhotla et al., 2019). Interestingly, 27 of the 124 EV cargo proteins secreted by the PC3 3D cultures were previously not registered in the Vesiclepedia database for PC3 cells (Figure 5b) (Kalra et al., 2012, Vesiclepedia accessed 26.7.2023). Of note, the Vesiclepedia database entries for PC3 cells were all obtained from 2D cultures and isolated using differential centrifugation, a method not optimal for mass spectrometry analyses due to low EV purity (Shao et al., 2018). The identification of 27 novel PC3 EV proteins in our study thus clearly shows that the culture conditions play a major role in EV protein loading.

Consistent with our hypothesis, we found that PC3 tumoroids secrete EVs with distinct protein contents before and after the invasive transition in 3D (Figure 5c–f). In the pre‐invasion samples (3D d2‐d8), we detected two unique proteins (Figure 5e), of which FGB was found in PC3 EVs for the first time (Kalra et al., 2012, Vesiclepedia accessed 26.7.2023). Intriguingly, the EVs secreted by invasive tumoroids (3D d10‐d14) contained several unique proteins promoting tumour growth and invasion, for example, SLC4A7, SLC12A2, FABP5, PRDX1, CD59 and CD73 (Figure 5c–e), showing that specific oncogenic signals are loaded in EVs upon invasion. These signals may promote different phases of metastasis, since EVs can modify the tumour microenvironment and have a role in pre‐metastatic niche formation during metastasis (Bhatta & Cooks, 2020; Zhang & Yu, 2019). When we chemically inhibited invasion of the 3D cultures, we detected an extensive reduction in the protein levels of CD59, CD73, VCL and ITGB4 in the d10‐d14 isolated EVs (Figure 6a–c). These results indicate that the invasive transition alters the protein profile of EVs.

CD59 has previously been detected in blood plasma (Yan et al., 2019) and urine EVs (Lu et al., 2009) from prostate cancer patients, as well as in blood plasma EVs from colorectal cancer patients (Dash et al., 2022), suggesting that CD59 is predominantly an EV cargo protein associated with later stages of cancer. Similarly, the levels of CD73 in EVs derived from head and neck squamous cell carcinoma (Lu et al., 2022; Theodoraki et al., 2018) or glioblastoma metastasis (Wang et al., 2021) patients have been shown to correlate with the severity of the disease, being higher at later stages and contributing to immunosuppression. Our findings also align with clinical data from 12 different cancer types, including prostate cancer, in the Human Disease Blood Atlas database, which identified increased levels of FABP5, PRDX1, CD59 and CD73 in the blood plasma of cancer patients (Álvez et al., 2023). Out of the 124 EV proteins we identified in 3D culture conditions, 24 were found in the Human Disease Blood Atlas. The alignment of our experimental findings with clinical data provides valuable insights into the potential clinical relevance of CD59 and CD73 in cancer, suggesting that these proteins could potentially serve as biomarkers of tumour progression.

In summary, this study demonstrates that the use of an ECM‐based 3D cancer invasion model was a prerequisite for the identification of novel EV cargo proteins that dynamically change according to the invasive status of the prostate cancer tumoroids. This approach is also applicable to cell types of different origins and can be combined with diverse downstream analyses of EVs. Thus, our EV production method holds a great potential for discovery of novel cancer‐derived EV components linked to tumour invasion and metastasis, which can serve as biomarkers for cancer.

## AUTHOR CONTRIBUTIONS

**Cecilia Jungarå**: Investigation; methodology; validation. **Sara H. Bengs**: Investigation; methodology; validation. **Jannica Roininen**: Investigation; methodology; validation. **Lea Sistonen**: Funding acquisition; project administration; resources; supervision; writing—review and editing. **Eva Henriksson**: Conceptualization; formal analysis; funding acquisition; investigation; methodology; project administration; resources; supervision; validation; writing—original draft; writing—review and editing. **Jens C. Luoto**:conceptualization; formal analysis; funding acquisition; investigation; methodology; validation; visualization; writing—originalDraft; writing—review and editing. **Leila S. Coelho‐Rato**: Investigation; methodology; validation. **John E. Eriksson**: resources; writing—review and editing.

## CONFLICT OF INTEREST STATEMENT

The authors declare no conflict of interest.

## Supporting information

Supporting Information

Supporting Information
