# Supplementary material for: Cancer cell invasion alters the protein profile of extracellular vesicles
Source: J Extracell Biol. 2023 Nov 27;2(12):e124. doi: 10.1002/jex2.124 (PMC11080925; doi:10.1002/jex2.124)
Supplement: Supplementary file 2 — Supporting Information [file JEX2-2-e124-s001.pdf]

SUPPLEMENTARY FIGURE 1

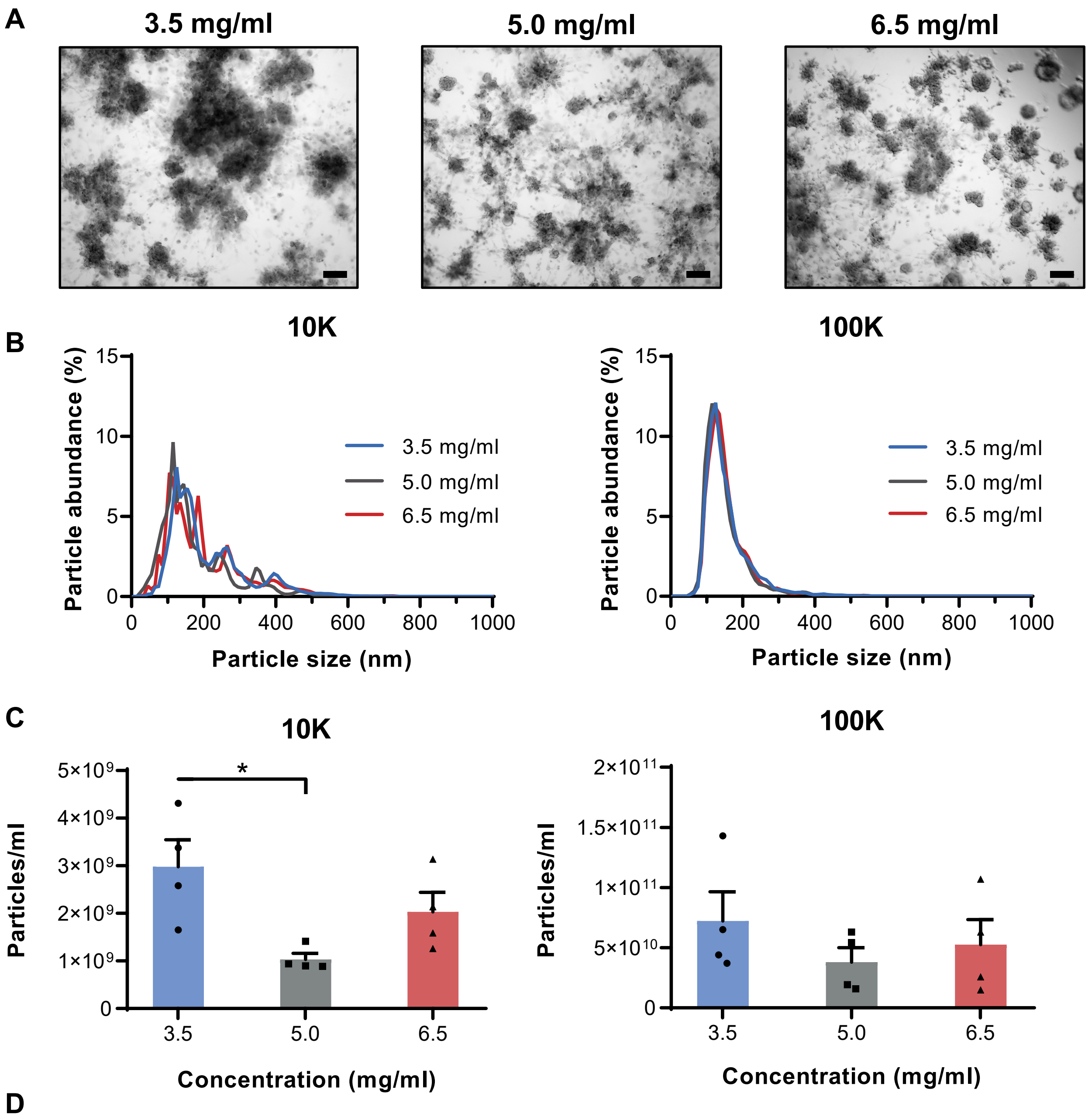

D

Matrigel concentration	10K Mean concentration (Particles/ml)	10K Mean size (nm)	10K Mode size (nm)	100K Mean concentration (Particles/ml)	100K Mean size (nm)	100K Mode size (nm)
**3.5 mg/ml**	2.98E+09	206	148	7.23E+10	155	120
**5.0 mg/ml**	1.03E+09	166	119	2.77E+10	146	115
**6.5 mg/ml**	2.03E+09	200	135	5.27E+10	149	129

Supplementary Figure 1. Matrigel concentration affects EV isolation efficiency. (A) Representative brightfield images of PC3 cells grown as in Fig 1 in Matrigel (3.5, 5.0 or 6.5 mg/ml) for 12 days. Scale bar 200 µm. **(B-D)** The size and concentration of particles isolated from conditioned media (day 10 to day 12) from PC3 cell cultures by 10K and 100K g centrifugation and analyzed by NTA. The used Matrigel concentrations were 3.5, 5.0, and 6.5 mg/ml. The data are presented as mean values of four independent experiments. **(C)** Significant differences were assessed by one-way ANOVA and Tukey's multiple comparison test, $^*p<0.05$, +SEM.

SUPPLEMENTARY FIGURE 2

Day	10K			100K		
	Mean concentration (Particles/ml)	Mean size (nm)	Mode size (nm)	Mean concentration (Particles/ml)	Mean size (nm)	Mode size (nm)
**2**	1.51E+09	160	95	4.42E+09	135	95
**4**	6.54E+09	182	105	1.44E+10	152	115
**6**	2.09E+09	163	135	1.83E+10	156	115
**8**	3.09E+09	152	125	9.53E+09	170	125
**10**	3.06E+09	160	115	2.86E+10	166	115
**12**	2.98E+09	211	125	7.23E+10	157	125

Supplementary Figure 2. Particle characteristics of EVs secreted by 3D grown PC3 cells. PC3 cells were cultured and EVs were isolated as described in Figure 1. The NTA data presents mean and mode values of 10K and 100K pelleted particles of four independent experiments.

SUPPLEMENTARY FIGURE 3

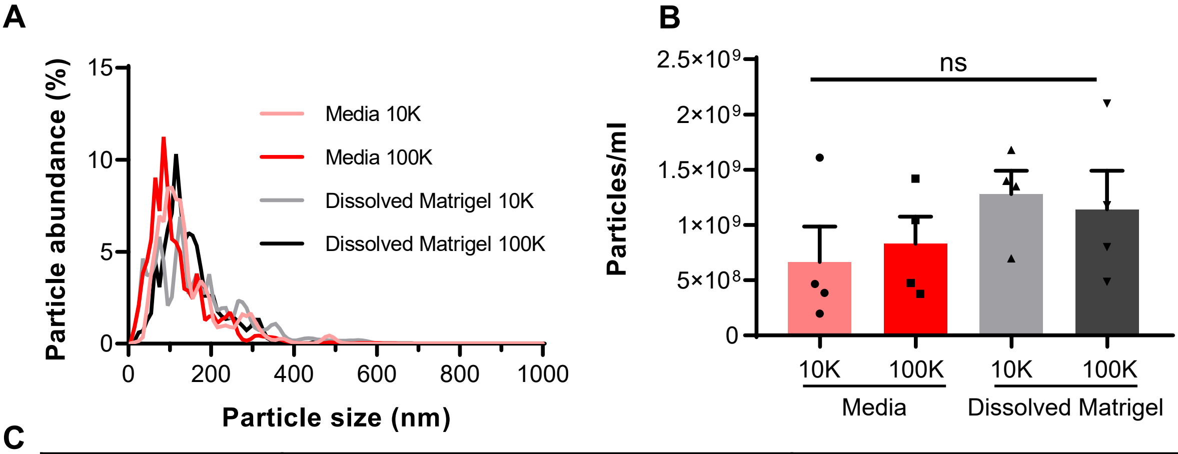

C

	10K			100K		
	**Mean concentration (Particles/ml)**	**Mean size (nm)**	**Mode size (nm)**	**Mean concentration (Particles/ml)**	**Mean size (nm)**	**Mode size (nm)**
**Media**	6.64E+08	158	93	8.30E+08	140	136
**Dissolved Matrigel**	1.14E+09	129	75	1.29E+09	149	141

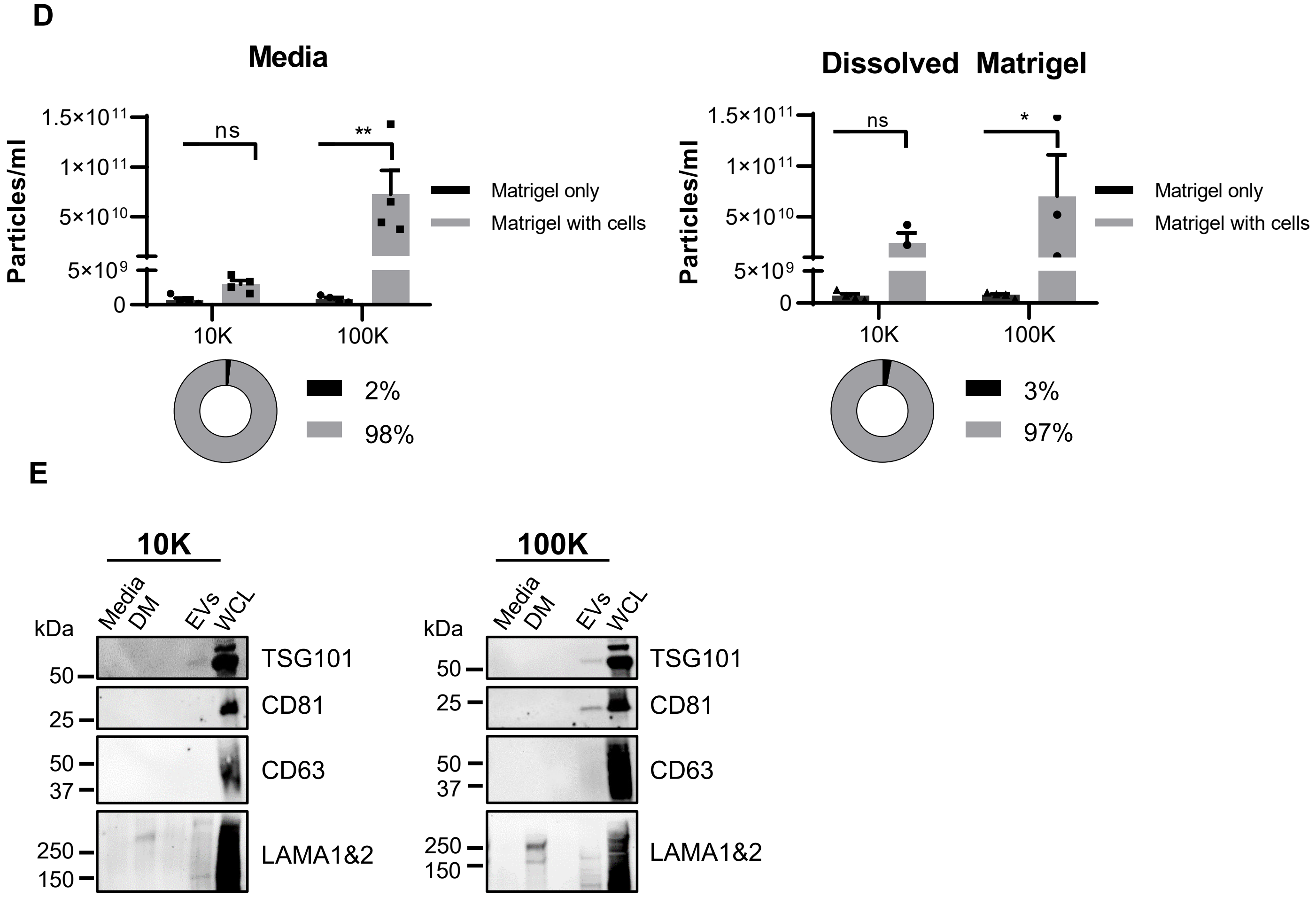

Supplementary Figure 3. Matrigel-derived particles do not impede EV isolation. Cultures without PC3 cells in 3.5 mg/ml of Matrigel were done as described in Figure 1. Media was collected at day 12 and dissolved Matrigel at day 14, and particles were isolated by differential centrifugation (10K and 100K g). The obtained results were compared to 3D cultures with cells. **(A-C)** NTA of media or dissolved Matrigel from conditions without cells. The data are presented as mean values of three independent experiments. **(B)** Significant differences were assessed by one-way ANOVA with Tukey's multiple comparison test, ns=not significant, *p<0.05, +SEM, n=4. **(D)** NTA of media or dissolved Matrigel from cultures with and without cells (days 10 to 12). The data are presented as mean values of three independent experiments. Significant differences were assessed by two-way ANOVA with Tukey's multiple comparisons test, ns=not significant, *p<0.05, **p<0.005, +SEM, n=4. Pie chart: particle amount comparison from media or dissolved Matrigel with and without cells (gray and black, respectively). The 10K and 100K sample particles were combined in this comparison. **(E)** Immunoblot analysis of EV markers TSG101, CD81 and CD63, and ECM components LAMA1 and LAMA2 in media and dissolved Matrigel (DM) from conditions without cells. EVs from conditioned media (EVs) and whole cell lysate (WCL) of 2D cultured PC3 cells were used as positive controls.

SUPPLEMENTARY FIGURE 4

A

3D d2-d8		3D d10-d14		3D Dissolved Matrigel		2D	
**Mean size (nm)**	**Mode size (nm)**	**Mean size (nm)**	**Mode size (nm)**	**Mean size (nm)**	**Mode size (nm)**	**Mean size (nm)**	**Mode size (nm)**
140	136	151	115	169	135	158	93

B

	3D d2-d8	3D d10-d14	3D Dissolved Matrigel	2D
**Ømin (nm)**	43	40	36	43
**Ømax (nm)**	235	283	353	283

Supplementary Figure 4. Non-invasive and invasive PC3 organoid cultures secrete EVs of similar size. (A) NTA results of EVs from non-invasive PC3 3D cultures at days 2-8 (3D d2-d8), invasive cultures at days 10-14 (3D d10-d14), dissolved Matrigel at day 14 (3D Dissolved Matrigel) and 2D cultures (2D). The data presents mean values of three independent experiments. **(B)** Minimum and maximum EV sizes (nm, diameter) observed by TEM from the EV samples. The data presents mean values of two independent experiments.
